# Neonatal sepsis in Iran: A systematic review and meta-analysis on national prevalence and causative pathogens

**DOI:** 10.1371/journal.pone.0227570

**Published:** 2020-01-24

**Authors:** Zahra Akbarian-Rad, Seyed Mohammad Riahi, Ali Abdollahi, Parisa Sabbagh, Soheil Ebrahimpour, Mostafa Javanian, VeneelaKrishnaRekha Vasigala, Ali Rostami

**Affiliations:** 1 Department of Pediatrics, Non-Communicable Pediatric Disease Research Center, Health Research Institute, Babol University of Medical Sciences, Babol, Iran; 2 Cardiovascular Diseases Research Center, Department of Epidemiology and Biostatistics, Faculty of Health, Birjand University of Medical Sciences, Birjand, Iran; 3 Department of Surgery, Faculty of Medicine, Tehran Medical Sciences, Islamic Azad University, Tehran, Iran; 4 Infectious Diseases and Tropical Medicine Research Center, Health Research Institute, Babol University of Medical Sciences, Babol, Iran; 5 Department of General medicine, Rangaraya Medical College, Kakinada, India; 6 Immunoregulation Research Center, Health Research Institute, Babol University of Medical Sciences, Babol, Iran; National and Kapodistrian University of Athens, GREECE

## Abstract

**Background:**

Neonatal sepsis is accounted for 30–50% of annual neonatal deaths in developing countries. We performed a systematic review and meta-analysis study to evaluate the national prevalence and identification of the etiological pathogens of neonatal sepsis in Iran.

**Methods:**

A comprehensive literature search was done on the national and international databases for studies published between 2000 and 2019. The DerSimonian and Laird random-effects model was used to calculate pooled prevalence estimates, with 95% confidence intervals (CIs). Subgroup analyses and meta-regressions regarding the gender, type of sepsis and time during were also performed. Data were extracted, analyzed, and presented according to PRISMA guideline.

**Results:**

Of 944 publications identified, 22 studies containing 14,683 neonates met the eligibility criteria. The pooled national prevalence of sepsis in Iran was 15.98% (95%CI, 11.96–20.46%; 1,367/14,683). Prevalence rate in boys (20.42%; 95%CI, 9.03–34.8%) was slightly higher than girls (18.5%; 95%CI, 7.4–32.8). A decreasing trend in prevalence of neonatal sepsis was found in recent years, although not statistically significant (c = -0.005; *P* value = 0.4). The most prevalent causative bacterial pathogens were *Enterobacter* spp. (23.04%), followed by *Klebsiella pneumoniae* (17.54%), coagulase-negative *Staphylococci* (14.06%), *Escherichia coli* (13.92%), *Pseudomonas aeruginosa* (12.67%), and *Staphylococcus aureus* (11.48%).

**Conclusion:**

Our findings showed a high prevalence of neonatal sepsis in suspected neonates, suggesting the need to implement preventive measures, routine assessment, and close monitoring of neonates. Also, *Enterobacter* spp. and *Klebsiella pneumoniae* were identified as the principal bacterial pathogens responsible for neonatal septicemia in Iran.

## Introduction

Sepsis is a serious cause of morbidity and mortality among neonates, killing approximately 3 million newborns each year [1]. The global burden of neonatal sepsis was measured as 2,202 per 100,000 live births [2]. Sepsis is a potentially life-threatening condition resulting from an extreme systemic immune response of the body to fight against an infection. This invasive infection, frequently bacterial, characterized by systemic signs of infection and isolation of bacteria from the bloodstream. Sepsis is one of the most important reasons for hospitalization of newborns in neonatal intensive care units (NICUs). Studies showed that neonatal sepsis is fatal and may quickly lead to septic shock and death if it's left untreated [3]. The therapy is often associated with an incontinent increase in antibiotic administration leading to development of antibiotic resistance.

Neonatal sepsis is categorized into either an early-onset neonatal sepsis (EOS) or late-onset sepsis (LOS) [4]. EOS is defined by bacteremia or meningitis occurring in newborns less than 3-days old [5]. EOS could be vertically transmitted infection that usually occurs as an ascending infection from the mother's cervix. Group B *Streptococcus* (GBS) is the leading cause of EOS [6] followed by *Escherichia coli* (*E*. *coli*) and *Listeria monocytogenes* [7]. Prematurity or low birth weight, birth asphyxia, prolonged rupture of membranes, and traumatic delivery are the major risk factors that contribute to the incidence of EOS [8]. LOS is defined as sepsis in infants during 4–90 days of life and could be caused either by vertically or horizontally transmitted infections [9]. The most important microorganisms involved in LOS include coagulase-negative *Staphylococci* (CoNS), *Enterobacter* spp., *Escherichia coli*, *Pseudomonas aeruginosa*, *Klebsiella pneumonia*, *Staphylococcus aureus* and *Candida albicans* [9, 10]. Some main risk factors associated with LOS are prematurity, low birth weight, poor hand hygiene, central venous catheters and prolonged mechanical ventilation [11].

In the past few years several studies were performed in Iran regarding the prevalence of neonatal sepsis in, although most of them were focused to limited geographical areas. Here, we performed a comprehensive systematic review and meta-analysis study to assess the prevalence of neonatal sepsis and identify the causative pathogens of the infection in Iranian neonates.

## Materials and methods

We used stepwise approach specified in the Preferred Reporting Items for Systematic Reviews and Meta-analyses guidelines [12] to perform of this systematic review and meta-analysis (S1 Checklist).

### Search strategy and selection criteria

Four major Iranian biomedical literature database servers (Iranmedex, Magiran, Irandoc and SID) and five international electronic databases (PubMed/MEDLINE, EMBASE, Scopus, Web of Science and Google Scholar) were searched for articles reporting the prevalence of sepsis in neonates and published between 2000 and 2019. The search terms utilized were “Iran”, “sepsis”, “septicaemia”, “bacteraemia”, “neonatal”, “neonate” and “neonates” (See S1 Text: Supplementary file for the details of the databases searches). We restricted the literature search to reports in English and Persian languages and human subjects. Bibliographies of the obtained studies and the relevant review articles were carefully evaluated to identify the studies that primarily cover the sepsis and its causative pathogens, but were not found in databases search. We only included observational original studies that had information on the prevalence of sepsis, reported data on the number of children with sepsis and the causative organisms, and neonatal sepsis cases diagnosed according to the standard guidelines (blood culture). Studies were excluded if they were done in adults, investigated only a single pathogen of sepsis, had sample sizes less than 50 cases, duplicate studies, reviews, letters, case-reports or case series without original data.

### Data extraction and quality assessment

After duplicates removal and screening of titles and abstracts, the full text of eligible studies were reviewed in depth by two independent reviewers (Z.A.R, and S.E). Data were extracted from all the eligible studies and compiled in Microsoft Excel spreadsheets. Inconsistencies were resolved by discussion and consensus with a third reviewer (A.R.). The following information were extracted from each study: first author last name, year of publication, study period, sample size, number of infected children, and number of bacterial pathogens. The quality of the studies was determined using Newcastle-Ottawa scale. Each item was scored for a maximum of six scores. Publications which scored 0–2, 3, 4 and 5 were classified as “unsatisfactory”, “satisfactory”, “good” and “very good”, respectively.

### Meta-analyses

The pooled prevalence of sepsis in Iranian neonates at a 95% confidence interval (CI) was estimated using the DerSimonian and Laird random-effects model (REM). The *I*^2^ statistic was used to assess the heterogeneity of the estimates. Subgroup analyses were performed according to the gender, type of sepsis (EOS and LOS) and time during. The pooled prevalence of each bacterial pathogen was also calculated using REM. Forest plots were used for showing of pooled prevalence rates by REM. We did not undertake an assessment of publication bias, as it is not relevant for the prevalence studies [13]. All analyses were performed using STATA v.13 (STATA Corp., College Station, Texas, USA). Statistical tests were significant if *P* value was <0.05.

## Results

**Fig 1:**
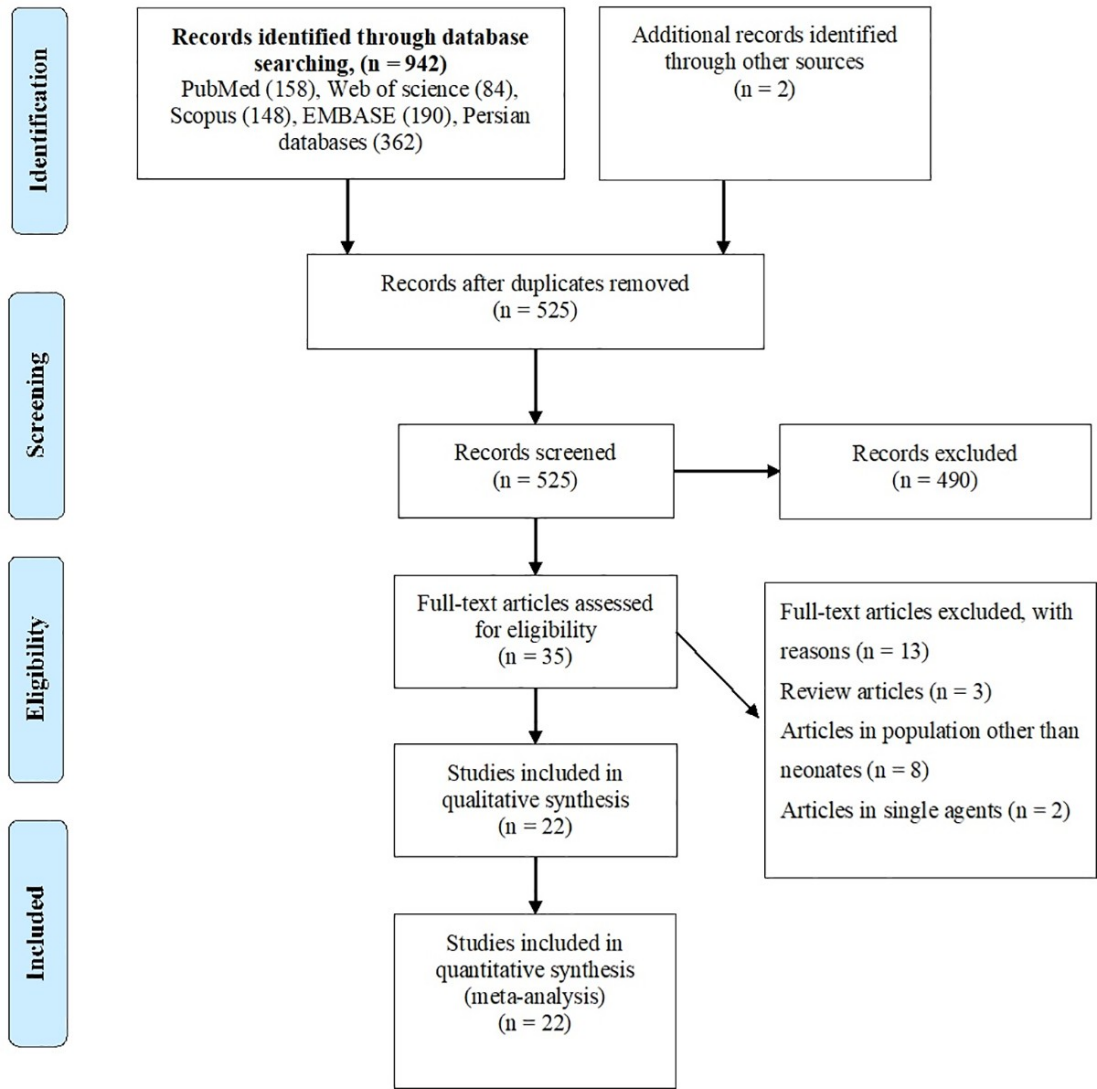
PRISMA flowchart showing the search and study selection strategy.

In the preliminary search, a total of 944 potentially relevant studies were identified through the search of the national and international databases, and 909 studies were excluded after duplicates removal and further review of titles and abstracts. Finally, 35 studies were reviewed in depth and 22 study papers [14–35] involving 14,683 neonates were found to be eligible for meta-analysis (Fig 1). The studies included in meta-analysis represented 12 different provinces covering all parts of the country. The province with the highest number of studies was Tehran (7/22). Five and seven studies had extractable data regarding the gender (including 847 boys and 664 girls) and type of sepsis (EOS and LOS). All studies used blood culture methods to detect sepsis, while information regarding the bacterial pathogens was reported in 19 studies. All these studies cover only the hospitalized neonates. Main characteristics of studies included are summarized in S1 File and Table 1.

**Table 1:** Main characteristics studies reporting prevalence of neonatal sepsis in Iran.

Authour	Publish year	Province	Number of neonates screened	Neonates with sepsis	Number of boys	Boys with sepsis	Number of girls	Girls with sepsis
**Malakan Rad et al. [14]**	2004	Isfahan	453	136	ND	ND	ND	ND
**Fesharaki Nia et al. [15]**	2004	South Khorasan	67	6	ND	ND	ND	ND
**Khosravi et al.[16]**	2004	Khozestan	200	10	123	8	77	2
**Movahedian et al.[17]**	2006	Isfahan	1680	111	ND	ND	ND	ND
**Salamati et al.[18]**	2006	Tehran	52	11	ND	ND	ND	ND
**Milani et al.[19]**	2007	Tehran	104	31	ND	ND	ND	ND
**Nikavar et al. [20]**	2008	Tehran	240	56	ND	ND	ND	ND
**Ghorbani et al.[21]**	2009	Guilan	298	31	ND	ND	ND	ND
**Torkaman et al.[22]**	2009	Tehran	114	53	ND	ND	ND	ND
**Hashemizadeh et al.[23]**	2009	Shiraz	578	78	ND	ND	ND	ND
**Shahian et al. [24]**	2010	Shiraz	208	90	115	54	93	36
**Monsef et al.[25]**	2010	Hamadan	417	105	239	60	183	45
**Dezfouli Manesh et al.[26]**	2011	Kermanshah	2175	90	ND	ND	ND	ND
**Aletayeb et al. [27]**	2011	Razavi Khorasan	3700	153	ND	ND	ND	ND
**Adib et al.[28]**	2011	Isfahan	69	20	ND	ND	ND	ND
**Karambin et al.[29]**	2011	Gilan	611	64	331	37	280	27
**Besharati et al.[30]**	2011	Khorasan	100	7	ND	ND	ND	ND
**Mosayebi et al.[31]**	2013	Tehran	1126	104	ND	ND	ND	ND
**Ahmadi et al.[32]**	2014	Khozestan	405	55	ND	ND	ND	ND
**Sharifi Yazdi et al.[33]**	2014	Tehran	216	55	ND	ND	ND	ND
**Khosravi et al.[34]**	2017	Tehran	70	17	39	8	31	9
**Afsharpaiman et al.[35]**	2019	Tehran	1800	84	ND	ND	ND	ND

**ND,** not determined

**Fig 2:**
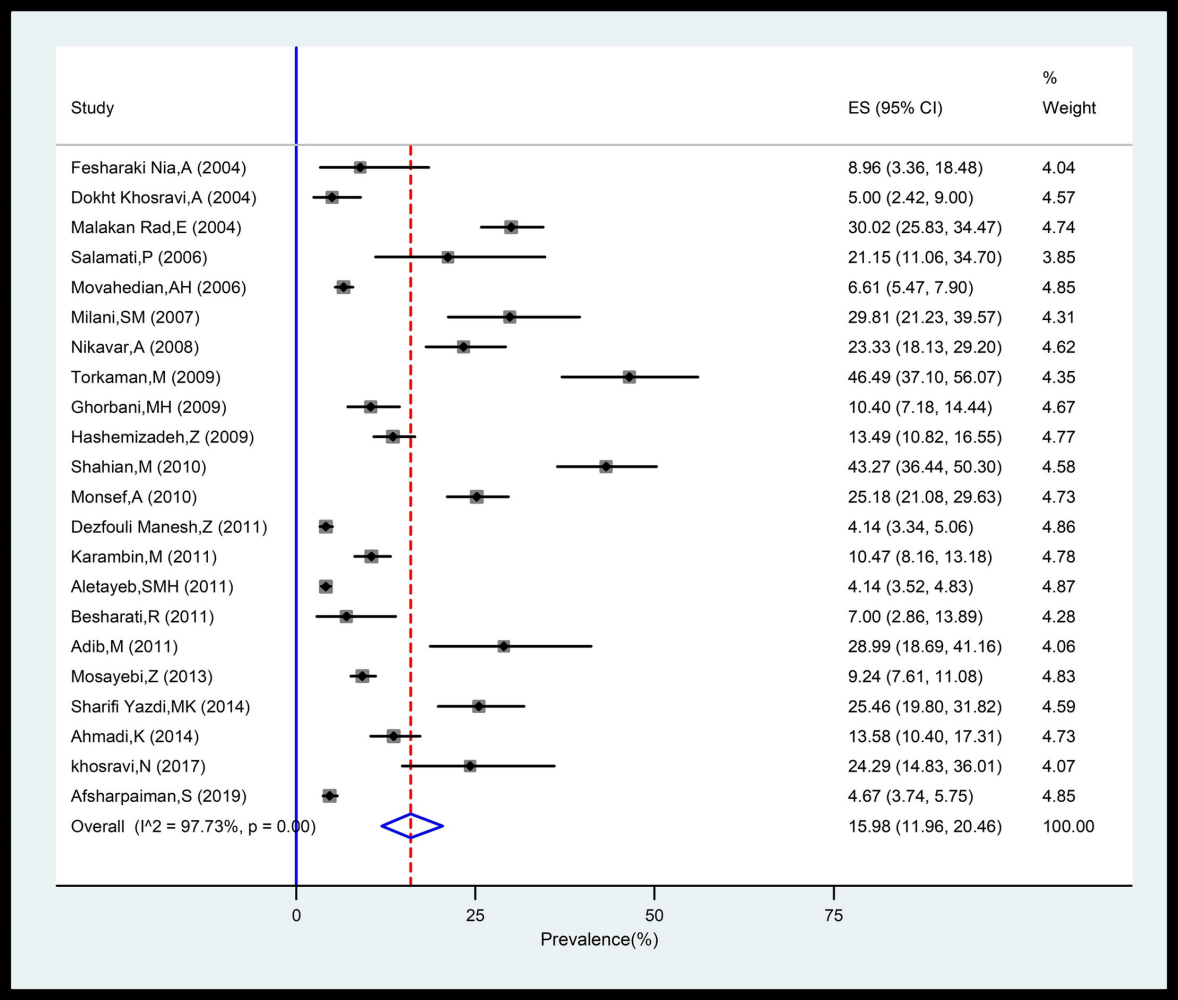
Forest plot of the prevalence of neonatal sepsis in Iran. ES: estimated prevalence.

**Fig 3:**
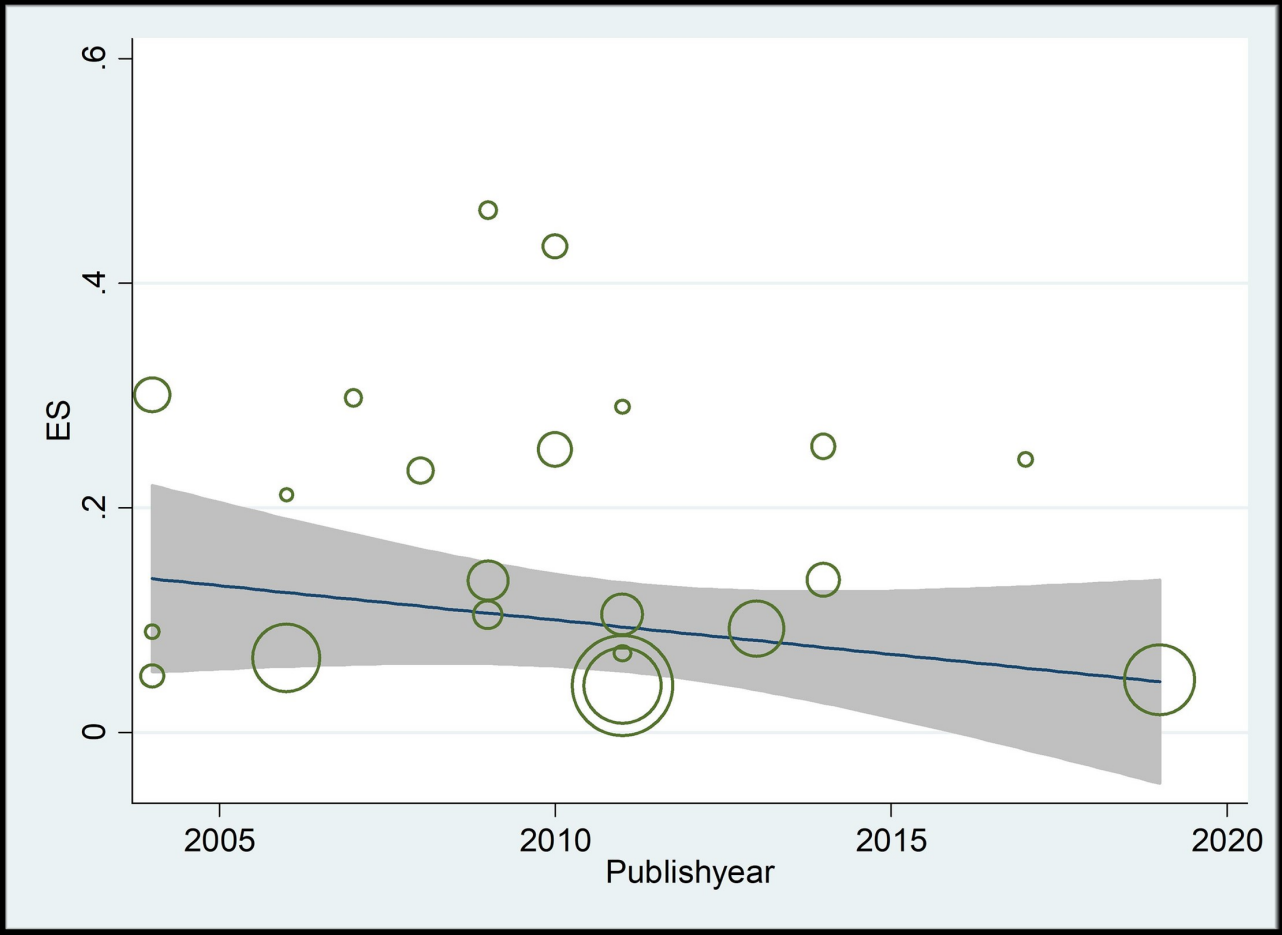
Meta-regression analysis of the prevalence of neonatal sepsis in Iran according to implementation years of screening showing a statistically non-significant decreasing trend in prevalence in recent years (c = -0.005; *P* value = 0.4).

Prevalence of sepsis among the studies was ranged from 4.14% (95% CI: 3.3–5.0%) in Kermanshah province in west of Iran to 46.49% (95% CI: 37.1–56.0%) in Tehran province in central part of Iran. The pooled national prevalence of sepsis in Iranian neonates (from 22 studies including 14,683 neonates) was 15.98% (95%CI, 11.96–20.46%; 1,367/14,683). Heterogeneity among studies was substantial (*I*^2^ = 97.7%, *P* < 0.001) (Fig 2). In a subgroup analysis with regards to type of sepsis, the pooled prevalence EOS and LOS were 10.96% (95%CI, 5.93–17.26%) and 6.85% (95%CI, 3.41–11.32%), respectively (Table 2, S1 Fig). With respect to gender, the pooled prevalence in boys (20.42%; 95%CI, 9.03–34.8%) was slightly higher than girls (18.5%; 95%CI, 7.4–32.8%), showing a trend that is not statistically significant (*P* value = 0.75) (Table 2, S2 Fig). Moreover subgroup analysis and meta-regression analyses showed a statistically non-significant but a decreasing trend in the prevalence of neonatal sepsis in recent years (c = -0.005; *P* value = 0.4; Table 2, S3 Fig and Fig 3).

**Table 2:** Prevalence of neonatal sepsis in Iran according to *a priori* defined stubgroups.

Variable/subgroups	Number studies	Number of neonates screened (total)	Number of neonates with proved sepsis	Pooled Prevalence (95% CI)	Heterogeneity *I*^2^ (%)
**Gender**					
**Boy**	5	847	167	20.42 (9.03–34.83)	95.0
**Girl**	5	664	119	18.51 (7.46–32.89)	93.7
**Type of sepsis**					
**Early onset sepsis**	7	8025	411	10.96 (5.93–17.26)	98.0
**Late onset sepsis**	7	8025	216	6.85 (3.41–11.32)	97.3
**Year**					
**2004–2008**	7	2796	361	16.46 (7.73–27.58)	97.1
**2009–2012**	11	9396	795	15.97 (10.31–22.57)	98.1
**2013–2019**	4	2491	211	15.50 (5.80–28.66)	97.3

**Fig 4:**
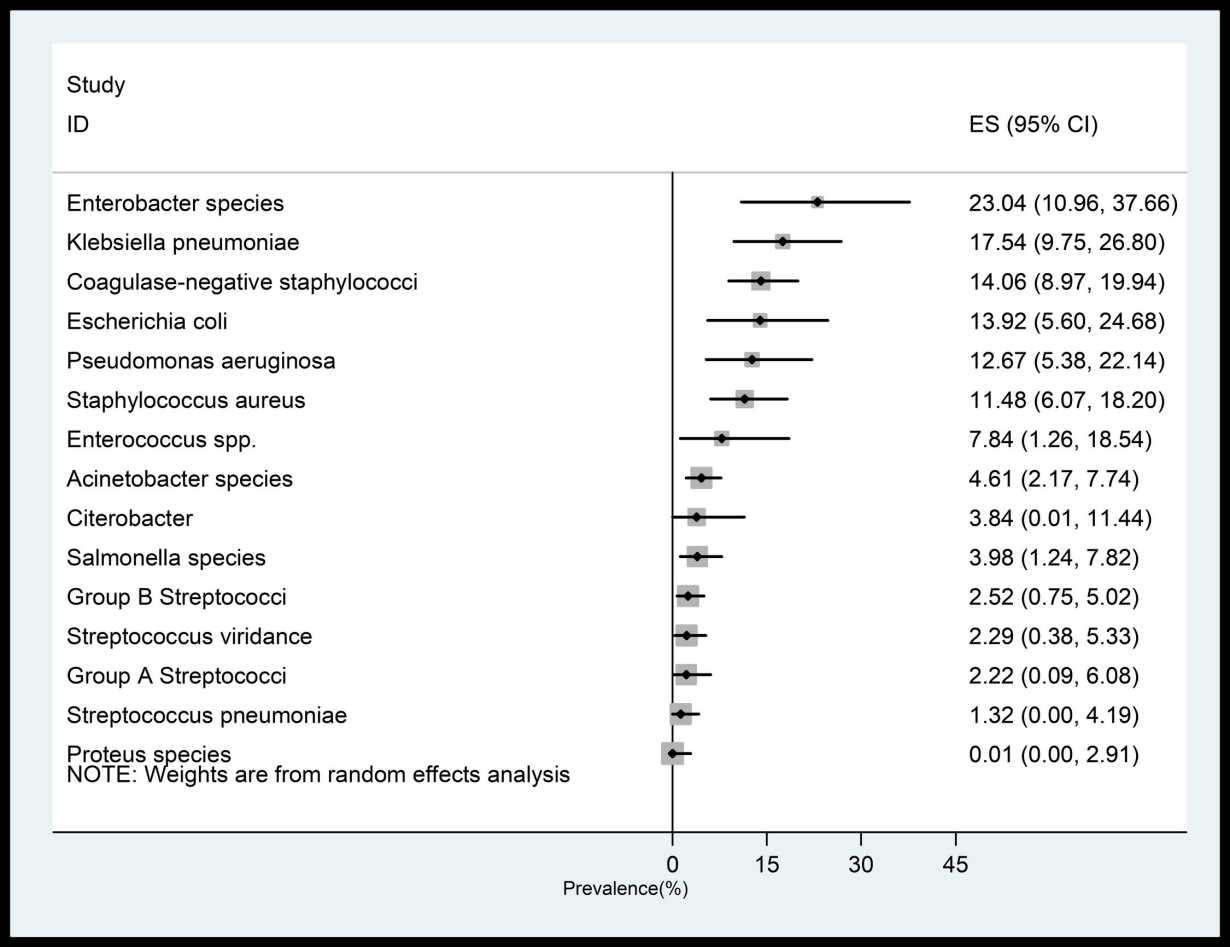
Forest Plot of the prevalence of each bacterial pathogens causing neonatal sepsis.

Among the bacterial pathogens causing the neonatal sepsis, our results indicated that the most prevalent causative pathogens belong to *Enterobacter* spp. (23.4%; 95%CI, 10.9–37.6%), followed by *Klebsiella pneumoniae* (17.5%; 95%CI, 9.7–26.8%), coagulase-negative *Staphylococci* (14.0%; 95%CI,8.9–19.9%), *Escherichia coli* (13.9%; 95%CI, 5.6–24.6%), *Pseudomonas aeruginosa* (12.6%; 95%CI, 5.3–22.1%), and *Staphylococcus aureus* (11.4%; 95%CI, 6.0–18.2%). Other minor bacterial pathogens were shown in Fig 4.

## Discussion

Neonatal sepsis continues to be a major health issue worldwide. A recent recommendation by World Health Organization (WHO) listed it as a life-threatening infection and a key healthcare priority for the coming decades [36, 37]. Therefore, studies that estimate the national and global prevalence data could be very useful to prioritize the control measures for this preventable disease. In this study, we report a country-level estimate of neonatal sepsis in Iran. Our estimates showed that the prevalence of sepsis in Iranian neonates is about 16%, with high variability in different provinces. The prevalence estimates were limited to hospitalized neonates and do not cover the entire population. Prevalence rate estimated in Iran is lower than those reported from Egypt (44%) [38], Tanzania (39%) [39], Cameroon (37%) [40], and higher than those reported from Oman (3.5%) [41], USA (5.16 per 1,000) [42], and Australia (0.5 per 1,1000) [43]. Also, the prevalence of sepsis in other studies from Nepal and India was reported 36% and 46.8%, respectively [44, 45]. These geographical variation could be attributed to preventive strategies adopted in each country, clinical criteria for sepsis diagnosis, differential sensitivity and specificity of the culture methods in different laboratories, health status of mothers during pregnancy, sanitary status in delivery section, and social-economic status in different countries [46, 47].

In consistent with previous studies in some parts of the world [38, 41, 42, 48], we found that prevalence of neonatal sepsis in boys is higher than girls. It is assumed that variation in genetic factors especially genes located in chromosome X may be responsible for these gender based differences [49]. In meta-regression analysis we have found a statistically non-significant but a decreasing trend in the prevalence of neonatal sepsis in recent years. This could be due to sustainable improvement of the sanitary conditions and efforts undertaken to improve mother and child health in Iran during last years [50, 51].

With respect to the causative pathogens of neonatal sepsis, our study indicated that *Enterobacter* spp. (23.0%) and *Klebsiella pneumoniae* (17.4%) were the most common organisms responsible for neonatal sepsis. Another large systematic review on causative pathogens of community-acquired neonatal sepsis in low- and middle-income countries demonstrated the most prevalent bacterial pathogens for neonatal sepsis as *S*. *aureus* (14.9%), *E*. *coli* (12.2%), and *Klebsiella* species (11.6%). However, these pattern were varied between different regions, as *S*. *aureus* and *Streptococcus pneumoniae* were most prevalent in Africa; *Klebsiella* in South-East Asia; *S*. *aureus* and *Klebsiella pneumoniae* in Europe and Western Pacific regions, and finally *Staphylococcus aureus* and *Haemophilus influenzae* in Americas region [52]. Moreover in consistent with our findings, results from another large systematic review indicated that *Enterobacter* species, *Klebsiella*, *Escherichia coli*, *Pseudomonas* species, and *Streptococcus pneumonia* were recognized as the most common bacterial pathogens of neonatal sepsis in the Middle East region [52]. In a country-level meta-analysis in China, Li et al reported that *Staphylococcus*, *Enterococcus*, *Escherichia*, and *Klebsiella* were found to be the most common organisms responsible for neonatal septicaemia in China [53]. This variation could also be explained by differences in geographical and climatic parameters in different regions.

The current study had some limitations that need to be considered when interpreting the results of this work. First, the majority of studies included were published in local journals and may have resulted in a publication bias. Second, although we have performed a comprehensive literature search, data from several provinces were limited, therefore the same estimates cannot be guaranteed from further studies that include these regions in the future. Third, we could not perform a detailed sub-group analyses other than what was presented in the manuscript, because the majority of studies included in the meta-analysis did not classify the neonatal sepsis cases based on type of delivery, birth weight and community-onset/hospital-associated infections. Forth, our meta-analysis demonstrated a substantial heterogeneity of neonatal sepsis prevalence across studies, thus making it difficult for a direct comparison. Nevertheless, our study performed on a large sample size and a rigorous methodology provides useful information for national authorities to prioritize prevention efforts and intervention programs to reduce the burden of neonatal sepsis in Iran. Our finding showed that about 16% of newborn infants hospitalized in Iran had sepsis, a statistic that is higher than those reported from developed countries. This high rate of neonatal sepsis can lead to several neonatal death annually, and call for further measures and interventions to control and prevent sepsis in Iranian neonates.

## Supporting information

S1 Checklist PRISMA checklist. (DOCX)

S1 Fig Subgroup analysis for the prevalence of neonatal sepsis in Iran according to type of sepsis (EOS and LOS). (TIF)

S2 Fig Subgroup analysis for the prevalence of neonatal sepsis in Iran according to gender. (TIF)

S3 Fig Subgroup analysis for the prevalence of neonatal sepsis in Iran according to publication year. (TIF)

S1 Text Details of the databases searches. (DOCX)

S1 File Main characteristics studies reporting prevalence of neonatal sepsis in Iran. (XLSX)
