# Supplementary figures and images for: Neonatal sepsis in Iran: A systematic review and meta-analysis on national prevalence and causative pathogens
Source: PLoS One. 2020 Jan 24;15(1):e0227570. doi: 10.1371/journal.pone.0227570 (PMC6980642; doi:10.1371/journal.pone.0227570)

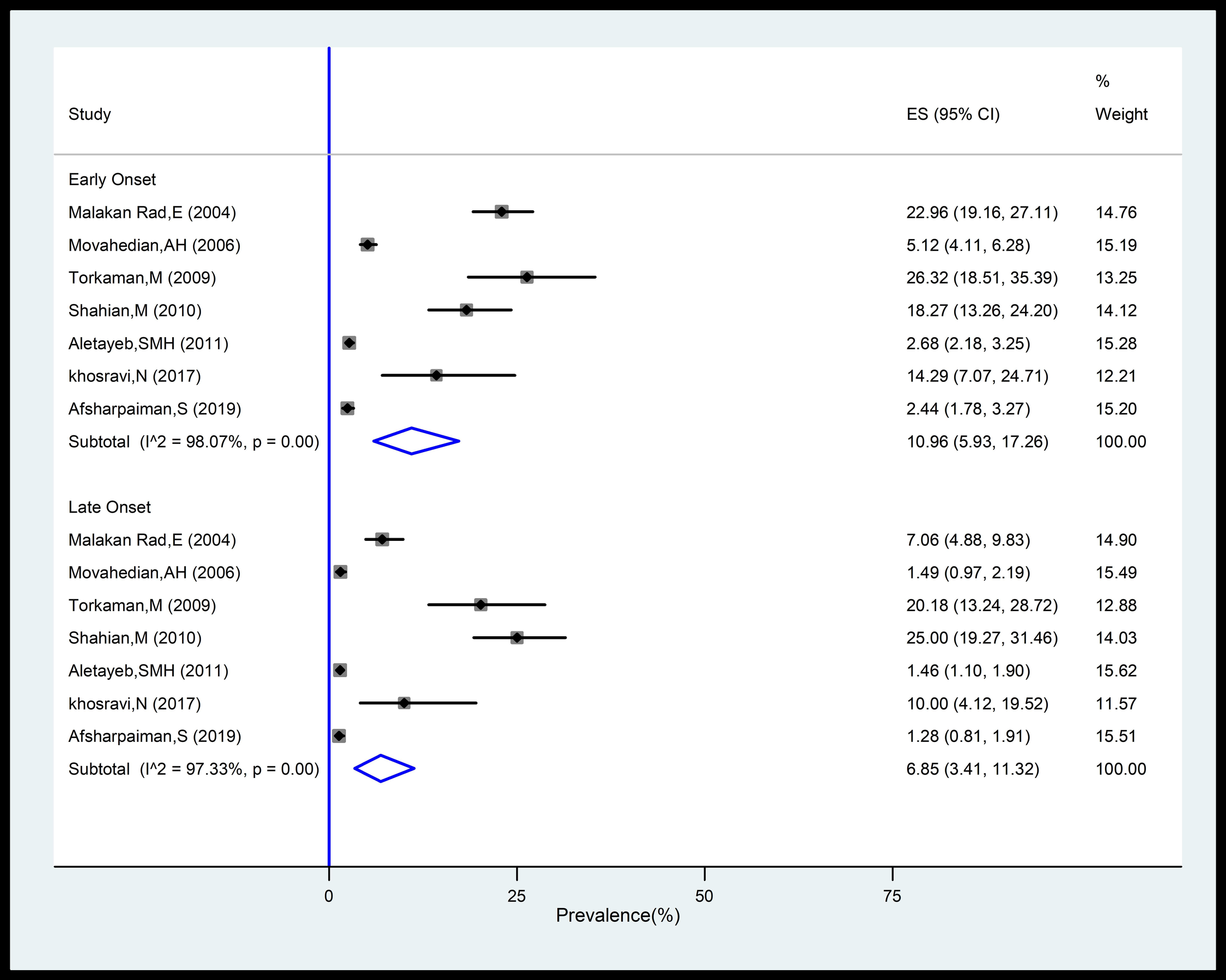

Supplement: S1 Fig — (TIF) [file pone.0227570.s002.tif]

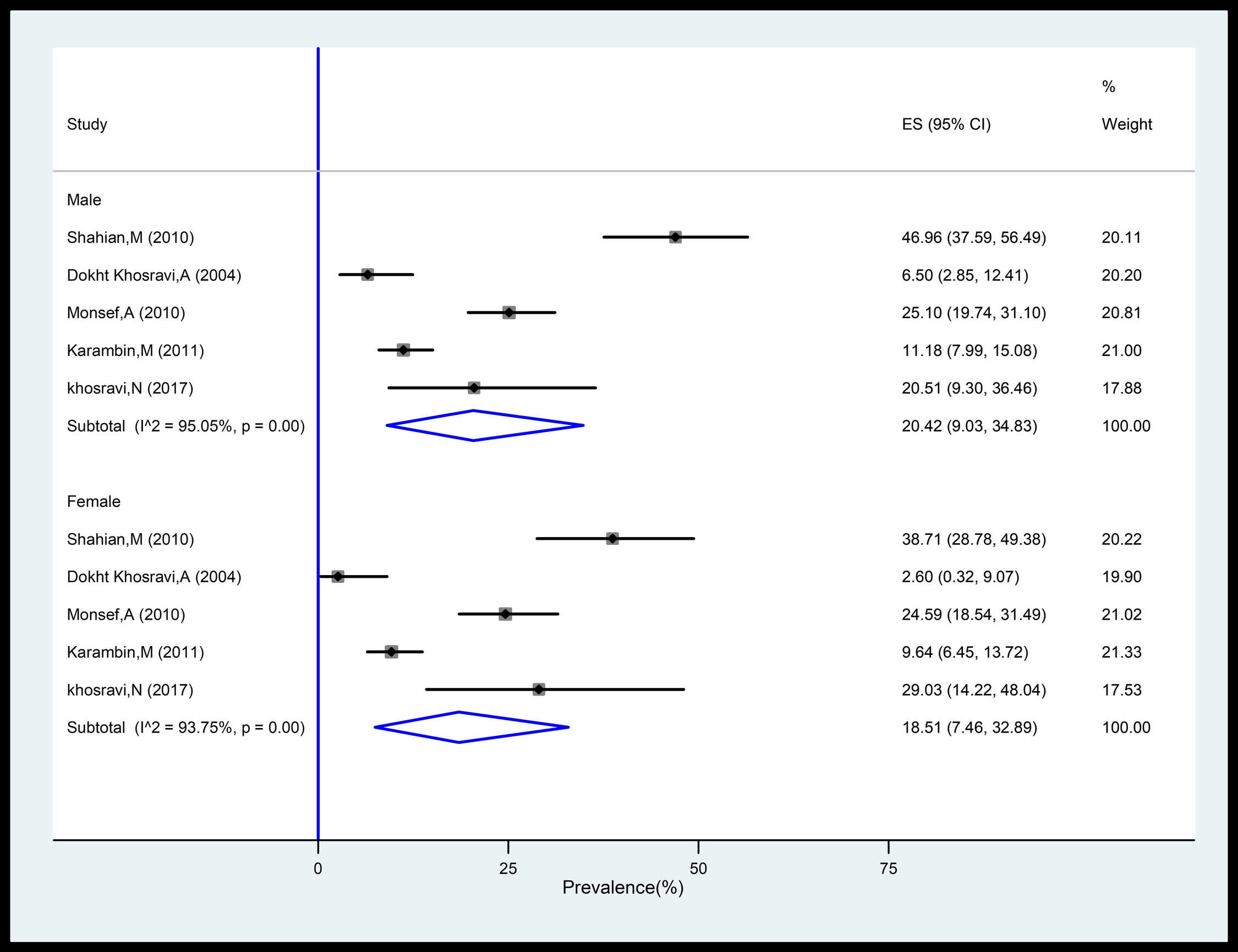

Supplement: S2 Fig — (TIF) [file pone.0227570.s003.tif]

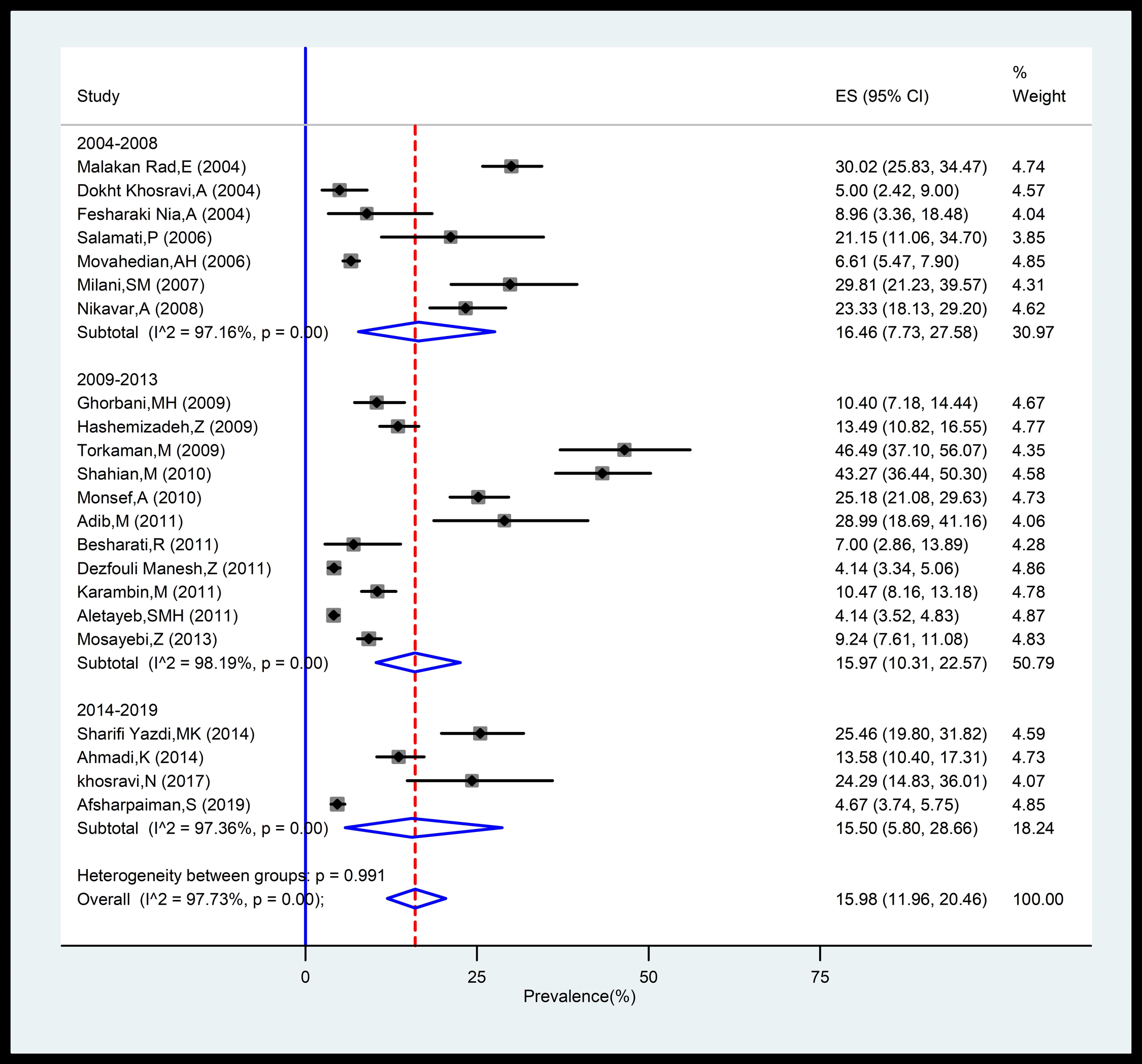

Supplement: S3 Fig — (TIF) [file pone.0227570.s004.tif]
