# Supplementary material for: Neonatal sepsis in Iran: A systematic review and meta-analysis on national prevalence and causative pathogens
Source: PLoS One. 2020 Jan 24;15(1):e0227570. doi: 10.1371/journal.pone.0227570 (PMC6980642; doi:10.1371/journal.pone.0227570)
Supplement: S1 Text — (DOCX) [file pone.0227570.s005.docx]

**PubMed= 158**

(("sepsis"[MeSH Terms] OR "sepsis"[All Fields]) OR ("septicaemia"[All Fields] OR "sepsis"[MeSH Terms] OR "sepsis"[All Fields] OR "septicemia"[All Fields]) OR ("bacteraemia"[All Fields] OR "bacteremia"[MeSH Terms] OR "bacteremia"[All Fields])) AND (("infant, newborn"[MeSH Terms] OR ("infant"[All Fields] AND "newborn"[All Fields]) OR "newborn infant"[All Fields] OR "neonatal"[All Fields]) OR ("infant, newborn"[MeSH Terms] OR ("infant"[All Fields] AND "newborn"[All Fields]) OR "newborn infant"[All Fields] OR "neonate"[All Fields]) OR ("infant, newborn"[MeSH Terms] OR ("infant"[All Fields] AND "newborn"[All Fields]) OR "newborn infant"[All Fields] OR "neonates"[All Fields])) AND ("iran"[MeSH Terms] OR "iran"[All Fields]) AND ("2000/01/01"[PDAT] : "3000/12/31"[PDAT])**= 158**

**Scopus=148**

TITLE-ABS-KEY ((*sepsis* OR *septicaemia* OR *bacteraemia*) AND (*neonatal* OR *neonate* OR *neonates*) AND (*Iran*)) **= 148**
